# Blood inflammatory biomarkers in participants with idiopathic epiretinal membrane: A retrospective case series study

**DOI:** 10.1097/MD.0000000000034225

**Published:** 2023-06-30

**Authors:** Guanghao Qin, Tiezhu Lin, Yue You, Mingxin Shang, Wei He, Emmanuel Eric Pazo

**Affiliations:** a He Eye Specialist Hospital, Shenyang, China; b Sinqi Eye Hospital, Shenyang, China.

**Keywords:** idiopathic epiretinal membrane, monocyte-to-lymphocyte ratio, neutrophil-to-lymphocyte ratio, platelet-to-lymphocyte ratio

## Abstract

The objective was to evaluate the levels of monocyte-to-lymphocyte ratio (MLR), neutrophil-to-lymphocyte ratio (NLR) and platelet-to-lymphocyte ratio (PLR) in patients with idiopathic epiretinal membrane (iERM). This retrospective case series study comprised of participants with iERM and participants with cataract. The values of MLR, NLR, PLR and from participants’ peripheral blood were assessed among groups. The best cutoff value of MLR, NLR, and PLR in iERM was found by performing a receiver operating characteristic curve analysis and determining the optimum cutoff value for each variable. In total, 95 participants with iERM were included in the study group, and 61 participants with senile cataract were included as controls. The lymphocyte count in the iERM group was significantly lower than the control group (1.69 ± 0.63 vs. 1.95 ± 0.53, *P* = .003). The monocyte count in the iERM group was significantly higher than the control group (0.39 ± 0.11 vs. 0.31 ± 0.10, *P* < .001). The area under the curve of MLR, NLR, and PLR in differentiating patients with IERM and controls was 0.782, 0.645, and 0.657, respectively, according to receiver operating characteristic. The best cutoff value of MLR was > 0.18, with sensitivity and specificity of 74.7% and 75.4%, respectively. The NLR was > 2.06, with a sensitivity and specificity of 50.5% and 83.6%, respectively. The PLR was > 95.89, with a sensitivity and specificity of 86.3% and 41.0%, respectively. The findings of this study suggest that systemic inflammation may be associated with iERM. IERM patients may be prone to have high MLR, NLR, and PLR values.

## 1. Introduction

Epidemiology studies show that the incidence of Idiopathic Epiretinal Membrane (iERM) increases as we age. It affects roughly 1.9% of those under 60 years, and 7.2% of individuals older than 60 years of age, with a slightly higher proportion of females being affected.^[1,2]^ iERM is a non-vascular membrane that covers the fundus and adjacent retina and is usually related to advanced age and posterior vitreous detachment (PVD).^[3]^ Participants can experience signs and symptoms such as reduced visual acuity, metamorphopsia, and monocular diplopia.

Although the exact cause of iERM is unclear, it is broadly accepted that anomalous PVD and inflammation are essential factors in its development.^[4,5]^ PVD may damage the internal limiting membrane of the retina and disrupt the blood-retinal barrier, activating the migration, proliferation, and aggregation of various retinal cells, as well as the secretion of a variety of growth and inflammatory factors that promote the formation of the anterior membrane.^[6,7]^ As an inexpensive and convenient marker of inflammation, monocyte-to-lymphocyte ratio (MLR), neutrophil-to-lymphocyte ratio (NLR), and platelet-to-lymphocyte ratio (PLR) have gained increasing attention in various disorders such as multiple types of cancers, COVID-19, Alzheimer disease, and acute coronary disease.^[8–13]^ Furthermore, these serum inflammatory biomarkers have been linked to the onset of ocular disorders such as dry eye disease, keratoconus, retinal vein occlusion, and retinal artery occlusion.^[14–18]^ While the role of NLR and PLR has been previously explored in iERM,^[19,20]^ the current study aimed to evaluate the probable relationship between MLR, NLR, and PLR serum inflammatory biomarkers in participants with iERM.

## 2. Material and method

### 2.1. Study design and participants

Participants diagnosed with iERM at the He Eye Specialist Hospital between January 2016 and October 2020 had their medical data evaluated retrospectively. The Ethics Committee at He Eye Specialist Hospital (Liaoning, China) approved this study, which followed the Declaration of Helsinki (IRB2022K004.01). As a control group, 61 age- and gender-matched cataract participants with normal fundus were enrolled.

ERM is defined as fibroblastic pre retinal membrane, which is diagnosed by an experienced retinal specialist using indirect ophthalmoscopy after dilating the pupil in conjunction with digital retinal imaging techniques using a 45° non-mydriatic camera (TRC-NW300, Topcon, Tokyo, Japan) and Cirrus HD-OCT 5000 (Carl Zeiss Meditec, Dublin, USA). Macular edema (ME) was diagnosed at the same time during the examination.

The study excluded participants who had any systemic diseases other than diabetes and hypertension, including infection, giant cell arteritis, any tumor, any blood disease, autoimmune disease, failure of the kidney and liver, heart disease, cerebrovascular disease, a history of surgery, drinking alcohol and smoking, family history of diabetic retinopathy, trauma, retinal artery or vein occlusion, glaucoma, or any other ocular disease. Anticoagulants, anti-inflammatory medications, anti-hyperlipidemia medications, and cancer-related treatments or medications were also excluded from this study.

### 2.2. Clinical evaluation

Intraocular pressure, slit lamp biomicroscopy, best corrected visual acuity, and indirect ophthalmoscopy following dilation of the pupil were all part of the thorough ophthalmological examination. Blood routine investigation (Auto-Blood Cell Analys BC-5180; Mindray, ShenZhen, China), as well as blood glucose and lipid profile analysis (Auto-chemistry Analys CS-1200; Mindray), were performed on overnight fasting blood samples of all participants in the same laboratory. The blood cell count, blood lipid profile, and fasting blood glucose level were all assessed. PLR, NLR, and MLR were manually calculated. Age, gender, weight, height, hypertension, diabetes, and body mass index was assessed.

### 2.3. Statistical analysis

The statistical analysis was carried out with the help of the SPSS statistical software (ver. 25.0; SPSS Inc., Chicago, USA). Among the descriptive statistics employed were the mean standard deviation (mean SD) and percentage. Categorical data was provided as percentages, and the Chi-square test was used to examine the data. The Kolmogorov–Smirnov test was used to determine whether or not the data was normally distributed. A Student *t* test was employed to determine whether or not there was any homogeneity of variance between the 2 groups. An examination of the receiver operating characteristics curve (ROC) was done to determine the sensitivity and specificity of baseline MLR, NLR, and PLR, and even the best cutoff value of iERM. The area under the ROC was used to predict validity. To determine statistical significance, a *P* value of less than .05 was employed, and the confidence interval was set at 95% CI.

## 3. Results

### 3.1. Patient characteristics

The final analysis of the study included 95 iERM participants (63 females and 32 males with a mean age of 65.91 ± 9.34 years) Forty-seven of the iERM participants had macular edema. Sixty-one senile cataract participants (40 females and 21 males with a mean age of 65.69 ± 6.67 years) were collected as control (Table 1).

**Table 1:** Baseline characteristics of participants.

	iERM (n = 95)	Control (n = 61)	*P* value
Age (yr)	65.91 ± 9.34	65.69 ± 6.67	.875
Gender, male (%)	32 (33.68)	21 (34.43)	.924
Hypertension, n (%)	21 (22.11)	12 (19.67)	.717
Diabetes, n (%)	9 (9.47)	6 (9.84)	.940
BMI (kg/m^2^)	23.82 ± 3.17	24.12 ± 3.32	.582

BMI = body mass index, iERM = idiopathic epiretinal membranes.

### 3.2. Parameters of the blood test

The parameters of the blood test are summarized in Table 2 in 2 groups. The study group’s lymphocyte count was considerably lower than the control group’s (1.69 ± 0.63 vs 1.95 ± 0.53, *P* = .003). The iERM group had a substantially higher monocyte count than the control group (0.39 ± 0.11 vs 0.31 ± 0.10, *P* < .001). The MLR, NLR, and PLR of the iERM group were considerably greater than those of the control group (all *P* < .001).

**Table 2:** The comparation of parameters of blood routine.

	iERM (n = 95) Mean ± SD	Control (n = 61) Mean ± SD	*P* value*
White blood cell count (10^9^/µL)	5.55 ± 1.34	5.69 ± 1.43	.540
Neutrophil count (10^9^/µL)	3.36 ± 1.03	3.25 ± 0.99	.501
Lymphocyte count (10^9^/µL)	1.69 ± 0.63	1.95 ± 0.53	.003
Platelet count (10^9^/µL)	214.17 ± 45.57	214.62 ± 50.21	.954
Monocyte count (10^9^/µL)	0.39 ± 0.11	0.31 ± 0.10	<.001
High-density lipoprotein, mg/dL	1.59 ± 0.34	1.62 ± 0.36	.492
Low-density lipoprotein, mg/dL	3.62 ± 0.96	3.74 ± 0.99	.466
Total cholesterol (mg/dL)	5.39 ± 1.04	5.53 ± 1.14	.431
Triglyceride (mg/dL)	1.74 ± 0.97	1.74 ± 0.83	.992
Fasting glucose (mg/dL)	5.71 ± 0.91	5.77 ± 0.83	.680
MLR	0.26 ± 0.11	0.16 ± 0.10	<.001
NLR	2.23 ± 1.00	1.70 ± 0.51	<.001
PLR	140.71 ± 51.97	113.94 ± 34.98	<.001

iERM = idiopathic epiretinal membranes, MLR = monocyte to lymphocyte ratio, NLR = neutrophil to lymphocyte ratio, PLR = platelet to lymphocyte ratio, SD = stand deviation.

* iERM compared to control group.

The iERM participants were subgrouped according to the presence or absence of macular edema, and there was no statistical difference in NLR, PLR, and MLR between participants with ME and non-ME (Table 3).

**Table 3:** The comparation of parameters of blood test in ME and non-ME groups.

	ME (n = 47) Mean ± SD	Non-ME (n = 48) Mean ± SD	*P* value*
Age (yr)	67.15 ± 6.98	64.69 ± 11.11	.200
White blood cell count (10^9^/µL)	5.72 ± 1.32	5.39 ± 1.35	.231
Neutrophil count (10^9^/µL)	3.44 ± 1.11	3.28 ± 0.94	.458
Lymphocyte count (10^9^/µL)	1.76 ± 0.69	1.63 ± 0.56	.303
Platelet count (10^9^/µL)	220.04 ± 46.17	208.42 ± 44.70	.216
Monocyte count (10^9^/µL)	0.40 ± 0.11	0.38 ± 0.11	.478
High-density lipoprotein, mg/dL	1.64 ± 0.39	1.53 ± 0.29	.097
Low-density lipoprotein, mg/dL	3.73 ± 1.06	3.52 ± 0.85	.300
Total cholesterol (mg/dL)	5.45 ± 1.07	5.32 ± 1.02	.547
Triglyceride (mg/dL)	1.78 ± 1.11	1.70 ± 0.82	.681
Fasting glucose (mg/dL)	5.77 ± 0.83	5.66 ± 1.01	.580
MLR	0.26 ± 0.12	0.26 ± 0.10	.861
NLR	2.26 ± 1.15	2.19 ± 0.84	.718
PLR	139.96 ± 51.38	141.44 ± 53.07	.890

iERM = idiopathic epiretinal membranes, ME = macular edema, MLR = monocyte to lymphocyte ratio, NLR = neutrophil to lymphocyte ratio, PLR = platelet to lymphocyte ratio, SD = stand deviation.

* iERM compared to control group.

**Figure 1.**
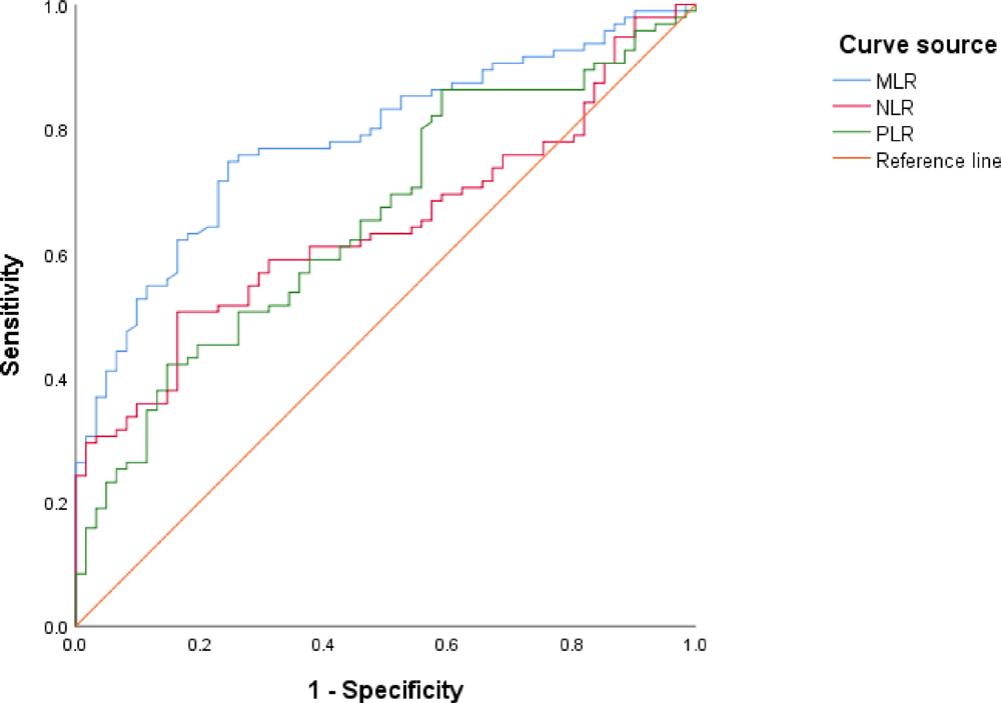
MLR, NLR, and PLR receiver operating characteristic curves for iERM predictors. iERM = idiopathic epiretinal membrane, MLR = monocyte-to-lymphocyte ratio, NLR = neutrophil-to-lymphocyte ratio, PLR = platelet-to-lymphocyte ratio.

The area under the curve of MLR, NLR, and PLR in differentiating patients with IERM and controls was 0.782, 0.645, and 0.657, respectively, according to ROC. The best cutoff value of MLR was >0.18, with sensitivity and specificity of 74.7% and 75.4% respectively. The NLR was >2.06, with a sensitivity and specificity of 50.5% and 83.6% respectively. The PLR was >95.89, with a sensitivity and specificity of 86.3% and 41.0% respectively (Fig. 1).

## 4. Discussion

iERM is a relatively common condition affecting the vitreous-macular junction. The patient has no clear history of eye disease, and the condition is most prevalent in the elderly. It has been established that the formation of iERM is associated with PVD, inflammation, and renin-angiotensin (RAS) system activation.^[4,21–23]^ According to previous reports, approximately 95% of clinically significant iERM occurs following vitreous body detachment.^[24]^ The iERM is primarily composed of retinal pigment epithelial cells and retinal glial cells (astrocytes and Müller cells).^[25]^ Histological studies have revealed that myofibroblasts, fibroblasts, clear cells, and macrophages may play roles in the occurrence and progression of iERM.^[25]^ Myojin et al^[26]^ examined the vitreous lavage fluid of participants with iERM (15 eyes) and found that pro-inflammatory genes were overexpressed (IL-6, GFAP, VEGFA, TGFB2, CXCL1, TNC, and RELA). Multiple interleukins (IL-1 IL-6, IL-13, IL-17) and TGF-β1, TGFβ2 interferon necrosis factor and other inflammatory factors are also involved in the formation of iERM.^[23,26]^ Furthermore, in the development of iERM, the RAS system is implicated.^[23,27]^ RAS can induce retinal vascular inflammation and mediate and regulate the factors involved in iERM fibrosis (FGF2, GDNF, NGF, TGF-β1).

Monocytes are pro-inflammatory during inflammation, whereas lymphocytes can help regulate inflammation.^[28,29]^ MLR has been used to investigate the staging and prognosis of COVID-19 and cardiovascular disease.^[9,13]^ In the current study, we found the monocyte count was significantly higher, and the lymphocyte count was significantly lower in the iERM participants. We used MLR to predict the development of iERM, and found good sensitivity and specificity. According to Joshi et al^[4]^ and Zhao et al,^[30]^ the major cell types in iERM are hyalocytes and müller cells. Hyalocytes are classified as macrophages (a kind of inflammation cells).^[31]^

Other 2 popular markers associated with the prognosis of systemic inflammatory diseases are NLR and PLR.^[32]^ Previous studies have established a link between NLR, PLR, retinal artery occlusion, retinal vein occlusion, and Keratoconus.^[15,18,33,34]^ Additionally, NLR and PLR were also used to classify the severity of dry eye.^[14]^ Though we found that iERM participants had significantly higher PLR and NLR values than the control group in the current study, that is inconsistent with the previous studies,^[33]^ in which the sensitivity or specificity was low. However, the lower sensitivity (52.6%) of NLR in predicting iERM was also reported in the study by Uzlu et al^[20]^ They attributed this difference to the small sample size.

Macular edema and macular holes could be caused by iERM traction. Triamcinolone acetonide (a long-acting adrenaline glucocorticoid) has the effects of anti-inflammatory, inhibiting cell proliferation, reducing the capillary permeability, stabilizing the retinal barrier, etc., and can reduce and control the progression of macular edema. Past studies have suggested that intravitreal injection of triamcinolone acetonide while removing the macular membrane can effectively reduce macular edema.^[35,36]^ That hint suggests that eyes with macular edema might be more associated with inflammation than usual. In addition, some clinical studies suggest that NLR and MLR can be used as inflammatory markers for diabetic macular edema.^[37,38]^ But we didn’t find any significant difference in MLR, PLR, and NLR between iERM participants with macular edema and those without macular edema in this study. The possible explanation is that macular edema in iERM eyes might be due to the physical traction, and inflammation might play a comparable role in the iERM with or without macular edema.

This study was limited by a number of factors. First, the vitreous fluid and postoperative macular membrane tissue tests were not available due to retrospective design. Second, we were unable to compare additional inflammatory markers simultaneously, such as interleukin-1 and TGF. However, to our knowledge, this is the first research to examine the link between MLR and iERM using a large sample size.

To summarize, it is possible that systemic inflammation may be associated with iERM. IERM patients may be prone to have high MLR, NLR, and PLR values. Additional laboratory studies on iERM’s mechanism are necessary to gain a better understanding of the relationship between iERM and blood biomarkers.

## Acknowledgments

We thank the participants in this study. This manuscript has not been published and is not under consideration for publication elsewhere.

## Author contributions

**Conceptualization:** Mingxin Shang, Wei He.

**Data curation:** Guanghao Qin, Yue You.

**Formal analysis:** Guanghao Qin.

**Investigation:** Tiezhu Lin, Wei He.

**Methodology:** Guanghao Qin, Wei He.

**Project administration:** Tiezhu Lin.

**Resources:** Mingxin Shang, Wei He, Emmanuel Eric Pazo.

**Software:** Yue You, Mingxin Shang.

**Supervision:** Mingxin Shang, Emmanuel Eric Pazo.

**Validation:** Yue You.

**Writing – original draft:** Guanghao Qin.

**Writing – review & editing:** Tiezhu Lin, Emmanuel Eric Pazo.
